# Challenging Recurrence and Management of Squamous Cell Carcinoma in the Calcaneal Region: A Case Report

**DOI:** 10.7759/cureus.59087

**Published:** 2024-04-26

**Authors:** Minh Phuong Tang, Thuy Thi Bich Nguyen, Huyen Thi Thanh Le, Hien Quang Nguyen, Nghia Minh Tran, Minh Huu Nhat Le

**Affiliations:** 1 General Surgery Department, Tra Vinh General Hospital, Tra Vinh City, VNM; 2 Internal Medicine Department, Tra Vinh General Hospital, Tra Vinh City, VNM; 3 Faculty of Medicine, University of Medicine and Pharmacy at Ho Chi Minh City, Ho Chi Minh City, VNM; 4 Cardiovascular Research Department, Methodist Hospital, Merrillville, USA; 5 Faculty of Medicine, Can Tho University of Medicine and Pharmacy, Can Tho, VNM; 6 International Ph.D. Program in Medicine, College of Medicine, Taipei Medical University, Taipei, TWN; 7 Research Center for Artificial Intelligence, College of Medicine, Taipei Medical University, Taipei, TWN

**Keywords:** recurrent tumor, nonmelanoma skin cancer, below-the-knee amputation, foot tumors, heel wounds, paraneoplastic syndrome, cutaneous neoplasms, squamous cell carcinoma

## Abstract

Squamous cell carcinoma (SCC) is the second most common type of skin cancer. As ultraviolet exposure represents an important risk factor, SCC commonly occurs on the face, lips, scalp, hands, and heels. The foot is an unusual location to manifest SCC. In this report, we present a case of a 44-year-old woman with severe local recurrence of SCC in the right heel, four years after an initial excision of a primary, small lesion. For various reasons, the patient did not visit the clinic for follow-up assessment during this period. Considering the extent of the lesion and infection risk, the affected leg was amputated at one-third of the lower leg. This case report underlines the importance of educating patients about the risk of SCC and assisting them in attending follow-up visits. In addition, adequate attention should be given to foot lesions with suspicious appearance. Early detection would minimize systemic risks, including metastasis and infection, and maximize preserved function after surgical intervention.

## Introduction

Squamous cell carcinoma (SCC) is on the rise as an important threat to global health. It is a subtype of non-melanoma skin cancers, alongside basal cell carcinoma (BCC). Historically, the prevalence of SCC was estimated at 20% of all skin cancer cases, second to BCC [1]. However, recent studies showed that SCC has been increasing rapidly. As tracked by the Global Burden of Disease Study, the annual incidence rates of this condition increased more than threefold from 1990 to 2017, the highest change among all neoplasms within their database [2]. Although basal cell carcinoma remains the most prevalent type of skin cancer, the gap is decreasing [3,4]. The disproportionate rise of SCC demands adequate attention given to screening, preventing, and treating this condition.

Reported risk factors for SCC include occupational sunlight exposure, immunosuppression, and chemical carcinogens [5-7]. Common locations for SCC to occur are skin areas exposed to the sun, such as the face, neck, scalp, forearms, hands, and shins [8]. SCC might arise from precancerous lesions, such as actinic keratoses, Marjolin's ulcer, and Bowen's disease [4,8-10]. The foot is not a common site of occurrence for SCC, with an incidence of 0.6-3.0% [11]. Nonetheless, SCC is the most common soft tissue cancer in the foot, capable of growing to significant sizes if untreated [12]. Attention should be given to any foot lesions with a chronic, non-healing progression [12]. In general, SCC in the foot tends to be locally invasive but carry a low metastasis risk [12-14]. Epithelioma cuniculatum is a possible manifestation of SCC, a slow-growing, well-demarcated, exophytic mass on the foot that exudes a foul odor [11,15]. This type of lesion could remain undiagnosed for many years, costing valuable time for treatment [16].

The treatment of choice for SCC occurring in the foot is surgical removal with a safe margin, at least 3-5 mm [11,12]. In case of recurrence, highly aggressive lesions that challenge the establishment of a safe margin, or malignant tissue found in the margin, more proximal amputation of the foot could be indicated [11,12]. Mohs micrographic surgery was proven to achieve better five-year overall survival than standard resection, at 86% versus 76% [17]. Other treatment options include curettage, cauterization, and cryotherapy [18]. The recurrence rate of SCC was reported to be 2.2-11%, with 80% of recurring disease happening within the first two years and 95% within five years of surgery [19]. Tumor depth is associated with local recurrence and metastasis, while tumor diameter exceeding 20 mm is correlated with disease-specific death [20]. Standard excision with wider surgical margins and postoperative margin assessment would minimize the risk of recurrence [12,21].

We present a case of a female patient with SCC in her right heel that recurred over four years after the initial wide excision of a small ulcer in the same location.

## Case presentation

A 44-year-old woman was admitted for a non-healing surgical wound on her right heel that exhibited abnormal growth and severe infection. Four years prior, she found a 2 x 3 cm ulcer on her right heel that did not improve over two months. The biopsy of the ulcer revealed SCC. Subsequently, a complete excision was performed, and the patient was referred to an oncology specialist for further care. There was no margin status after excision. However, hindered by the COVID-19 pandemic and personal reasons, she discontinued follow-up visits. The resulting surgical wound did not heal well and was ulcerated. Six months after the surgery, a mass grew around the wound, enlarging over time, causing local pain and limiting ankle movement. In the last six months leading up to the time of admission, the mass was infectious and exudative, and the patient suffered from on-and-off fever, significant functional impairment of her right foot, weight loss of 4 kg, and loss of appetite.

**Figure 1:**
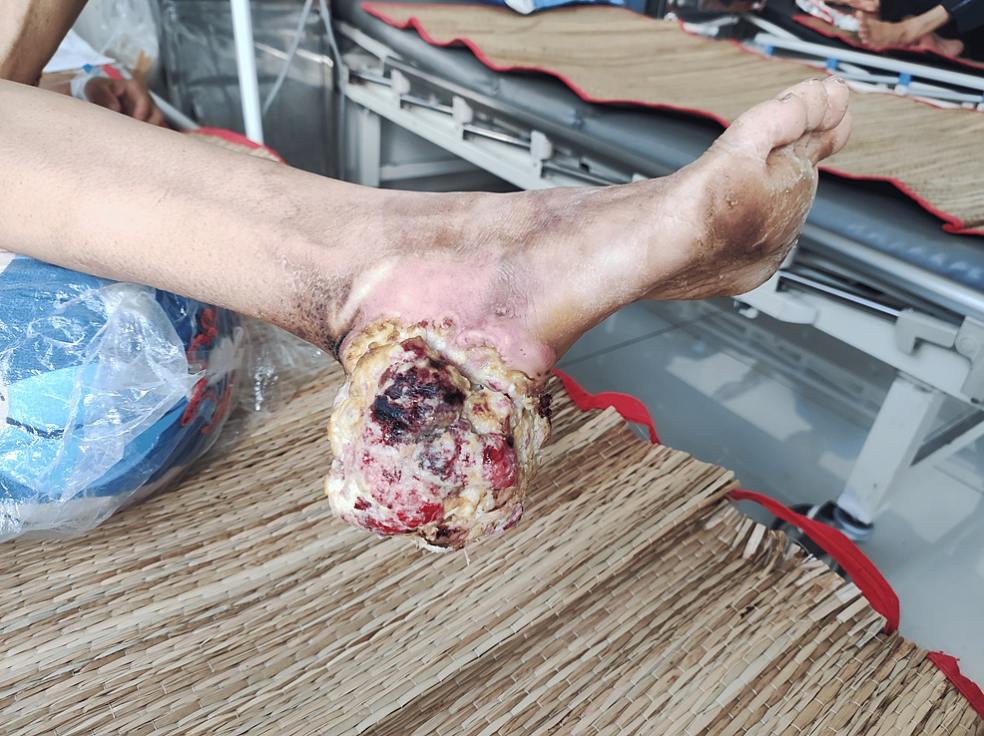
Squamous cell carcinoma lesion on the right heel of the patient

**Figure 2:**
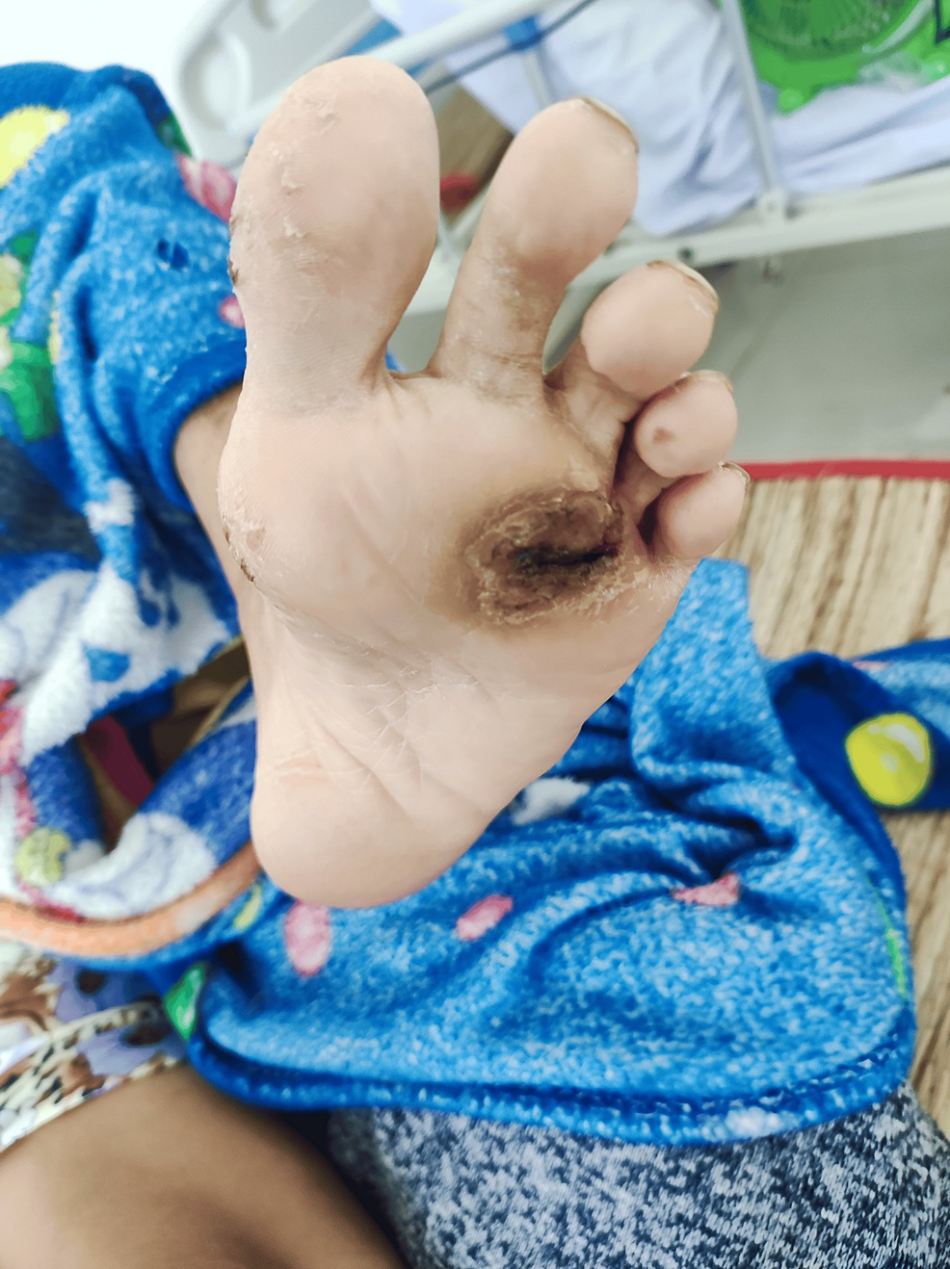
Shallow ulcer on the left sole

At the time of admission, her vital signs showed a mild fever of 38 degrees Celsius, a heart rate of 90 bpm, blood pressure of 110/70 mmHg, and a respiratory rate of 18 bpm. Her weight and height were 53 kg and 155 cm, respectively. Her medical history included hypertension and type 2 diabetes. The sole of her left foot had a shallow ulcer near the third and fourth digits, with a brownish and dry surface. Her right heel was encompassed by an extensive, tender growth, measuring approximately 8 x 6 x 6 cm (Figure 1). The surface of the mass was hypergranulated, with sparse areas of fibrosis and necrosis. Infectious exudates and mild serosanguineous drainage were noted. The margin between the mass and her skin was erythematous with pseudomembrane. A malodorous odor emitted from the wound. Movement of her right ankle was reduced. The sole of her left foot had a dry, brownish, nontender, shallow ulcer measured 4 x 4 cm, near digits III and IV (Figure 2). The pulses on both legs were regular in strength. Further examination revealed no abnormalities in peripheral lymph nodes and intact neurological function.

**Figure 3:**
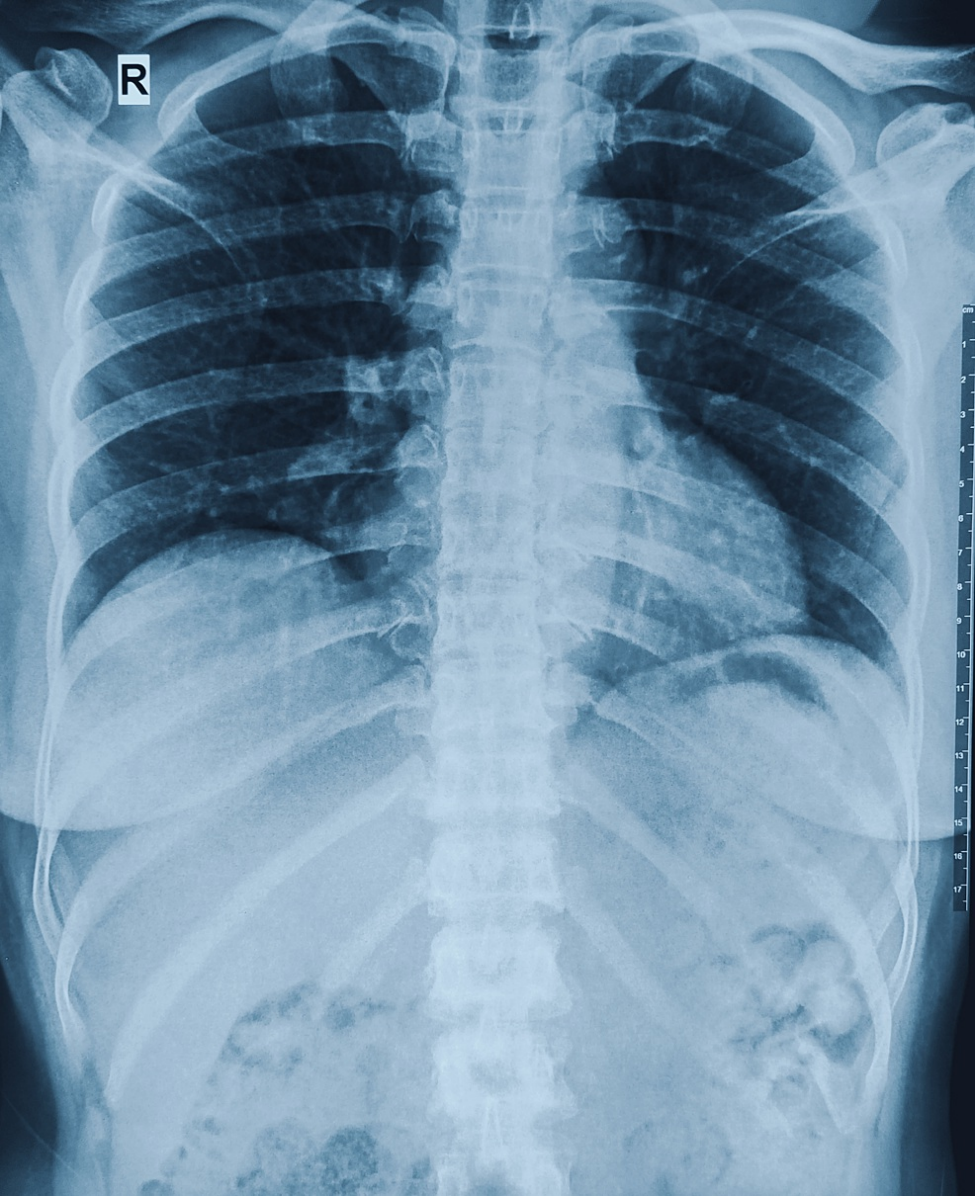
Chest X-ray showed no abnormalities.

**Figure 4:**
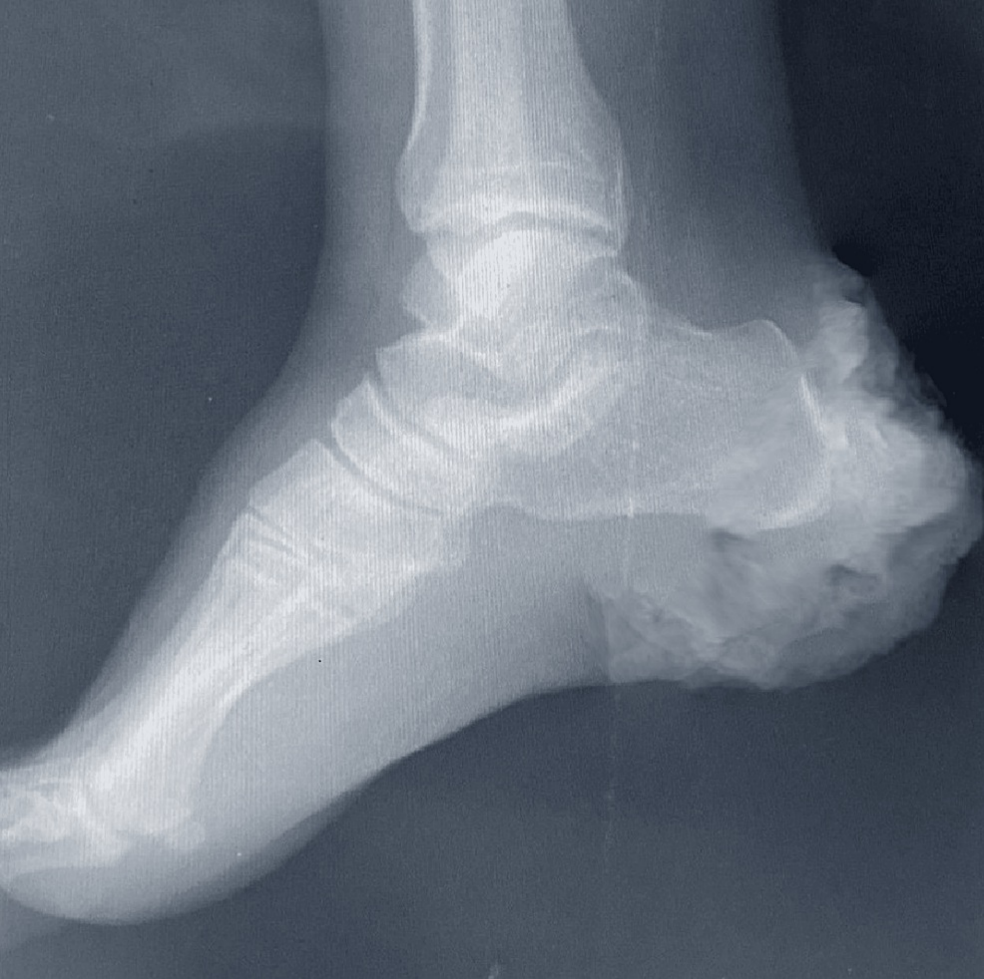
X-ray imaging of the right heel, with cutaneous lesion visible and no evidence of bone invasion.

Her laboratory test results showed an elevated leukocyte count at 20.7 K/µL, with a 91% neutrophil percentage, The C-reactive protein level was normal at 1.74 mg/dL. Her platelet and erythrocyte count were regular, while the hemoglobin level was slightly low at 10.5 g/dL. Her chest X-ray was unremarkable (Figure 3), while an X-ray of the right heel did not find evidence of calcaneus invasion (Figure 4). PET-CT was not available at our local hospital. A microbial culture was performed for one blood sample and one exudate sample from her right heel growth. The blood culture was negative, while the exudate culture found *Klebsiella pneumoniae *sensitive to piperacillin-tazobactam, ceftazidime, imipenem, and meropenem.

A multidisciplinary team meeting was held with the participation of oncology specialists. Upon taking into consideration the severely infected status of her right foot, the recurring nature of the SCC lesion, and the possibility of systemic infection, amputation was evidently a solution with minimal risk. After being informed about the status and risks of the lesion, the patient and her family chose amputation over preserving surgery or conservative treatment. 

**Figure 5:**
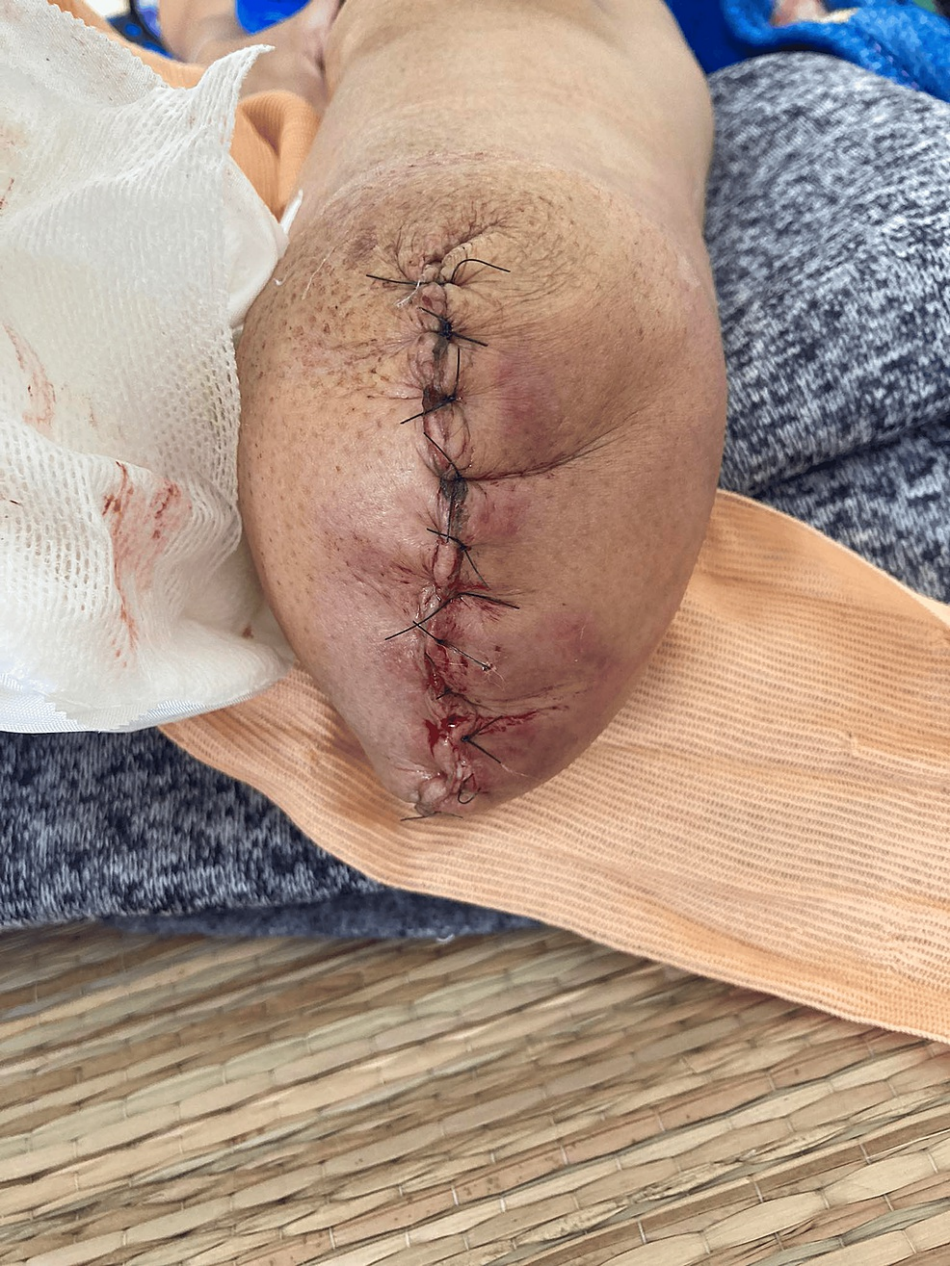
Sutured stump at the lower one-third point of the right lower leg.

The patient was prepared for amputation of the lower one-third of her lower right leg and her right foot. The surgery successfully excised soft tissue and bone structure to form a stump at the lower one-third of the right lower leg without any complications (Figure 5). Gross investigation during surgery recorded local invasion of bone structures. Intravenous cefepime and metronidazole were chosen to prevent post-surgical infection. The patient recovered without incident.

The histopathology result of the punch biopsy taken from the growth during surgery revealed SCC. There was mild hyperplasia of the epidermis and intradermic clustered dysplastic cells with enlarged nuclei and hyperchromasia. Margin biopsy and sentinel lymph node biopsy were not performed. Because of financial difficulties, the patient and her family members disagreed to pursue further laboratory and imaging screening methods for metastasis after the surgery. The patient and family members were made aware of the increased metastasis risk and the crucial importance of attending follow-up visits. The patient was referred to oncology specialists for outpatient monitoring and rehabilitation.

## Discussion

In this case, the most important concern is the risk of metastasis and recurrence. According to the National Comprehensive Cancer Network, this case carries a high risk for recurrence, with the presence of at least two risk criteria: a tumor larger than 20 mm in an extremity and a recurrent tumor [17]. Tumor thickness is another factor in predicting metastasis risk, with a 15% chance of metastasis for a tumor thicker than 6 mm [22]. Raval et al. reported a set of criteria for identifying high-risk SCC cases, which included late diagnosis, large tumor size of over 2 cm, deep invasion of over 4 mm, indistinct clinical border, rapid growth, local metastases, and poor differentiation [11]. SCC lesions developed over chronic ulcers, as in our case, also carried a higher risk of metastasis [12]. As this patient met many criteria for a high risk of metastasis, this possibility should be the foremost concern in the follow-up phase after surgery.

Surgery is generally agreed to be the first-line treatment for SCC [11,14,23]. Wide local resection is often the chosen option for primary lesions [11]. However, this type of surgery might carry an increased recurrence risk, between 19% and 75% [24]. Mohs micrographic surgery is another option, with reduced recurrence risk at 12.9-16% and minimal distant metastasis risk [24,25]. Amputation could be indicated in the case of extracutaneous involvement, such as calcaneus invasion [26]. In our case, the severity and recurrence of the lesion prompted the decision to amputate the affected foot. Although computerized tomography (CT) scan and magnetic resonance imaging (MRI) were not available because of resource and financial constraints, gross examination during the amputation surgery revealed local bone invasion. The risk of recurrence after amputation was reported to be 7.7% [24].

In addition, Burtonet al. suggest that combining adjunct radiotherapy with surgery could enhance outcomes significantly compared to surgery alone, potentially reducing the recurrence rate by half and boosting the five-year disease-free survival rate by an estimated 35% [22]. The National Comprehensive Cancer Network further recommended chemoradiotherapy in local high-risk, very high-risk, and metastatic SCC cases [21]. However, other studies have raised concerns over the risk of increasing disease malignancy with radiotherapy [26,27]. As SCC in the foot is an elusive clinical phenomenon, there is limited evidence concerning radiotherapy and chemoradiotherapy for this specific subtype. For SCC in the head and neck, Kiyota et al. recommended postoperative radiotherapy at 1.0 to 2.0 Gy daily or postoperative chemoradiotherapy with high-dose cisplatin [28].

In our case, the patient refused comprehensive investigation and management, possibly due to her socioeconomic situation. Consequently, we had to decide on the optimal course of action, which involved monitoring for signs of recurrence and metastasis without prescribing chemotherapy or radiotherapy after surgery. This introduces some limitations to our case report. First, we were unable to obtain consent to perform a CT scan and MRI on the patient. Because of this, distant metastasis could not be ruled out. Second, a biopsy of the left foot lesion was not available at the time. Finally, we could not ensure the patient's adherence to follow-up assessments.

In general, her chief concern was the severe condition of her right foot, and the prognosis of her illness, both of which were improved by amputation of the affected leg below the knee. She expressed optimism about rehabilitation post-surgery. The length of hospitalization and cost of treatment were within the capacities of her family. After a discussion with the healthcare team, she understood the importance of long-term monitoring and would make time for regular checkups.

## Conclusions

Our case highlighted a rare clinical phenomenon, cutaneous SCC of the heel. The features of the case suggested a high risk of recurrence and metastasis; however, the patient's socioeconomic constraints limited our capacity to address this issue. The existing lesion on the right heel was infectious and threatening sepsis, and amputation was the chosen solution, in accordance with medical literature recommendation in cases of recurring SCC in the leg. More recently, Mohs micrographic surgery was proven to achieve better long-term prognosis than standard resection. Adjuvant chemoradiotherapy could be prescribed when there was a high risk of metastasis, although evidence was limited, especially for lower-extremity SCC. We proposed periodic surveillance to detect metastasis early, a solution to balance protecting the health of the patient and their socioeconomic limitations.
